# Can Financial Support Reduce Suicide Mortality Rates?

**DOI:** 10.3390/ijerph16234797

**Published:** 2019-11-29

**Authors:** Ryo Kato, Motohiro Okada

**Affiliations:** Department of Neuropsychiatry, Division of Neuroscience, Graduate School of Medicine, Mie University, Tsu 514-8507, Japan; ryo-kato@clin.medic.mie-u.ac.jp

**Keywords:** suicide mortality, Japan, prefecture, financial support

## Abstract

After the launch of governmental financial support for the development of a regional suicide prevention programme, ‘Emergency Fund to Enhance Community-Based Suicide Countermeasure’ in 2009, suicide mortality rates in Japan have decreased from 25.7 (in 2009) to 16.5 (in 2018) per 100,000 population. Therefore, to explore the effects of governmental financial support on suicide mortality rates in Japan, the present study determined the relationship between the trends of empirical Bayes standardised Mobile Ratio of suicide mortality ratio in all 47 Japanese prefectures (EBSMR-RR) and the execution amounts of 10 sub-divisions of ‘Emergency Fund to Enhance Community-Based Suicide Countermeasure’ using stepwise multiple regression analysis. The female EBSMR-RR was only significantly/inversely related to the municipal ‘development of listeners and leaders’, whereas male EBSMR-RR was significantly/inversely related to prefectural ‘enlightenment’, and ‘intervention models’, but significantly/directly related to prefectural ‘personal consultation support’. The present findings suggest the inverse relationship between financial support and the suicide mortality rates in Japan. Furthermore, the independent factors in the reduction of suicide mortality rates between males and females provide important information for planning a scientifically evidence-based and more cost-effective regional suicide prevention programmes.

## 1. Introduction

**Figure 1:**
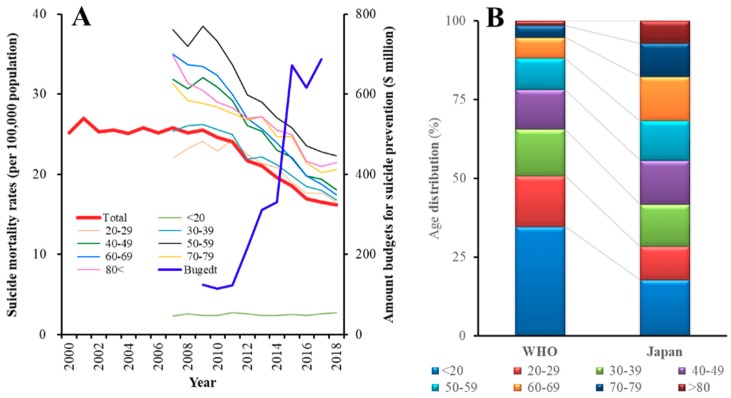
(**A**) Trends of age-dependent Japanese suicide mortality rates and government budgets for comprehensive suicide prevention programmes. Left and right ordinates indicate suicide mortality rate (per 100,000 population/year) and budget amounts ($million/year), respectively. (**B**) Comparison of age-distribution between World Health Organisation (WHO) world standard population model and Japan. Ordinate indicates age-distribution rates (%). Japanese age distribution was significantly different from WHO world standard population model by Mantel–Haenszel test (χ^2^ = 52.1, *p* < 0.05).

In the past two decades, Japan has experienced one of the world’s highest suicide mortality rates. Indeed, Japan achieved the dubious distinction of having the highest suicide mortality amongst the Organisation for Economic Co-operation and Development countries [1]. Until the early 1990s, suicide mortality amongst Japanese males (17–18 per 100,000 population) was lower than that of European males. However, following the collapse of the asset bubble in 1991 and immediately following the 1997 Asian economic crisis, suicide mortality in Japan rose to more than 30,000 deaths in 1998 (at maximum, 40.1 deaths per 100,000 males in 2003) [2,3]. In the face of this public health crisis the Japanese government enacted the ‘Basic Act on Suicide Prevention’ [4] in 2006, and the ‘General Policies for Comprehensive Measures against Suicide’ in 2007 [4]. In 2012, the government introduced a ‘Second Comprehensive Suicide Prevention Policy’ and approved a budget of over $216 million per year to support suicide prevention activities (Figure 1A) [2,4,5]. Based on this financial support, the comprehensive suicide prevention programme, which gathered the most up-to-date information on suicide prevention research, introduced a system for the early detection of high-risk individuals, targeted medical service delivery to such individuals, and supported suicide survivors. Indeed, following this programme of government action, Japanese suicide mortality rate fell, from around 26 per 100,000 between 2000 and 2009 to 16.5 per 100,000 in 2018 (Figure 1A) [3,4]. 

According to these two policies, the Japanese government contributed funds between 2009 and 2014 to prefectures and municipalities to strengthen regional programmes in the form of the ‘Emergency Fund to Enhance Community-Based Suicide Countermeasure’ of the Ministry of Health, Labour and Welfare (MHLW) in order to encourage a comprehensive suicide prevention programme [4,6]. Indeed, since 2009, the year the emergency fund started, the Japanese suicide mortality rate improved, from around 25.5 per 100,000 between 1998 and 2009 to 16.5 per 100,000 in 2018 (Figure 1A) [3,4,7]; however, the effects of the ‘Emergency Fund Enhance Community-Based Suicide Countermeasure’ on the suicide mortality rate remain to be clarified.

Previous research found that a socioeconomic crisis is a contributing risk factor in Asia [8]. In the 30 years, known as the “lost decades” of economic stagnation that followed the collapse of the asset bubble, the socioeconomic pattern of male suicides in Japan reversed [9]. While the lowest suicide rates were reported for managerial and administrative workers in 1975, by 2005 the rates for this demographic were only second to those for service workers. In contrast, in 1975, production workers, sales workers, and clerical workers had higher suicide mortality rates than managerial workers, but, by 2005, the rates of these groups had declined to less than half that of managerial workers [9]. Despite these findings, the detailed mechanisms of the rise in suicide mortality rates in Japan have yet to be clarified. The differences in trends between Japan and South Korea suggest that financial support plays an important role in effective suicide prevention. Until 2003, the South Korean suicide mortality rate was lower than that of Japan. However, after 2006 and the introduction of the ‘Basic Act on Suicide Prevention’, the Japanese suicide mortality rate dropped below that of South Korea [2]. Indeed, in 2017, financial assistance for suicide prevention programmes in South Korea was approximately $16.8 million, when compared to $687 million in Japan (Figure 1A) [2,4,5,10].

Based on achievements in reducing the suicide mortality rate in the past decade, in 2017, the Japanese government introduced a new national programme, the ‘Revised Basic Act on Suicide Prevention’ [4], and, in 2019, the ‘Law Concerning the Promotion of Research and Utilisation of Results to Contribute to the Comprehensive and Effective Implementation of Suicide Prevention’ [4,11]. This new programme is based on previous comprehensive actions and evidence-based support for regional (prefectural and municipal) suicide prevention programmes [11]. Regional factors also play an important role in suicide mortality. Prefectures with lower socioeconomic status, as measured according to average yearly income and average savings, have been found to have a higher suicide mortality risk [12]. In 2002, in advance of other prefectures, Mie Prefecture, which traditionally had one of the lowest suicide mortality rates in Japan, introduced a suicide prevention programme that included a “leader and listener training programme” [13]. Prior to the introduction of this programme between 1996 and 2002, Mie Prefecture had a suicide mortality rate that was similar to most other prefectures [14]. However, during the active years of the programme, and, until 2016, Mie’s suicide mortality rate was lower than other prefectures [3,4,13]. However, at a recent meeting concerning Mie’s suicide prevention measures, it was suggested that the regional programme should be revised and further developed, in response to the prefecture currently having a suicide mortality rate higher than the national average. In 2017, Mie Prefecture had a suicide rate of 17.9 per 100,000, when compared to a national rate of 16.5, and, in 2018, it had a rate of 18.1, when compared to a national rate of 16.2 [3]. 

Therefore, the present study determined the relationship between the distribution of suicide prevention budgets and suicide mortality trends based on the 2017 Revised Basic Act and the 2019 law mentioned above in order to develop a more cost-effective and evidence-based regional suicide prevention programme for Mie Prefecture [11].

## 2. Materials and Methods 

### 2.1. Data

The study used age-, gender-, and prefecture-specific data on suicide mortality rates between 2009 and 2018, obtained from the Ministry of Health, Labour, and Welfare (MHLW) and the Statistics Bureau of the Ministry of Internal Affairs and Communications (SBMIAC) of Japan [3,15]. Annual age-, gender-, and prefecture-specific suicide data were derived from the basic data on suicide in the region (MHLW), while population exposure (denominator) was obtained from the basic resident register (SBMIAC) [15]. Data on each prefecture’s suicide prevention programme budget were derived from the ‘Emergency Fund to Enhance Community-Based Suicide Countermeasures’ of the MHLW. These data were published between 2009 and 2014 [4,6]; further data were unavailable from the MHLW [6]. Although the 10-year survey period (2009–2018) does not exactly coincide with the six-year funding period (2009–2014), previous research found that the suicide mortality rate response in Japan needed several years following exposure to severe risk factors [2,16]. A statistical analysis of the relationship between prefectural suicide mortality rates and the emergency fund was conducted based on suicide prevention programme regional execution amounts (REAs) despite the inconsistency between survey and funding periods. REAs consisted of 10 sub-divisions, including prefectural and municipal “personal consultation support programme”, “telephone consultation support programme”, “development programme of leaders and listeners”, “enlightenment programme”, and “intervention model programme”.

### 2.2. Summary of Emergency Fund to Enhance Community-Based Suicide Countermeasures

The ‘Emergency Fund to Enhance Community-Based Suicide Countermeasures’ is composed of support for five independent prefectural and five municipal regional programmes [6]: 

“Personal consultation support programme”, which “utilize professionals, such as lawyers, social workers, public health nurses cooperating widely and contributing to suicide prevention” and “provide consultation on daily life, such as unemployment, bankruptcy, and multiple debt problems, which are risk factors for suicide, and consultation on health factors; regional comprehensive support consultations are organized” [6]. 

“Telephone consultation support programme”, which enhances the facilities that are necessary for telephone support, including telephone number sharing and 24-hour free support conducted by prefectural, municipal, and private organizations [6]. 

“Development programme of leaders and listeners”, which organizes workshops for human resources training to provide consultation support for persons at high risk of suicide, persons who have attempted suicide and bereaved family members [6]. 

“Enlightenment programme”, which enhances public/social support awareness for high-risk persons while using publicity via newspapers, TV, radio, distribution of pamphlets, symposiums, lectures, etc. [6]. 

“Intervention model programme”, which supports survey and support programmes for high-risk persons, carried out independently by prefectural or municipal organizations [6].

Municipalities submitted their regional suicide prevention programmes to their prefecture. Prefectures submitted their prefectural programmes along with municipal programmes to MHLW. MHLW allocated funds to each prefecture, including budgets for prefectures and municipalities [6]. Suicide prevention programmes of prefectures and municipalities were independent of each other. 

### 2.3. Standardisation of Suicide Mortality Rates

Usually, studies of international trends of suicide mortality analyse standardised death rates while using the WHO world standard population model [17]; however, the age distribution in Japan is significantly different from that in the model (χ^2^ = 52.1, *p* < 0.05) (Figure 1B). Therefore, in the present study, the annual suicide mortality rate was calculated based on the standard mortality ratio (SMR), which has been used in previous studies to adjust for age and gender factors that affect suicide mortality rates [18]. SMR was calculated by dividing the observed number of suicides by the expected number of suicides. The male and female ratios were calculated separately within eight age subgroups: under 20, 21–30, 31–40, 41–50, 51–60, 61–70, 71–80, and over 80 years. Once the national rate was calculated, the population of each prefecture multiplied it in order to obtain the expected number of suicides in each prefecture. SMR can be affected by large variances in less populated prefectures when compared to larger prefectures, as a result of variation in the population size. Therefore, SMR indices were standardised while using the empirical Bayes standardized mobile ratio (EBSMR) [19], using the EB estimator for the Poisson/gamma model ver. 2.1 to standardise suicide mortality rates in 47 prefectures (National Institute of Public Health, Wako, Japan) (https://www.niph.go.jp/soshiki/gijutsu/download/ebpoig/index_j.html).

### 2.4. Statistical Analysis

The least squares method was used to analyse time-dependent trends in the reduction of suicide mortality ratios in each prefecture (EBSMR-RR) by BellCurve for Excel ver. 3.2 (BellCurve, Tokyo, Japan). SPSS for Windows version 26 (IBM, Armonk, NY, USA) was used to conduct linear and stepwise multiple regression analyses of the relationship between EBSMR-RR and REAs to investigate the relationship between prefectural suicide reduction trends (EBSMR-RR) and financial support for regional prevention programmes (‘Emergency Fund to Enhance Community-Based Suicide Countermeasures’). Multicollinearity was suspected if the variance inflation factor (VIF) value was greater than 10.

## 3. Results

### 3.1. 2009–2018 EBSMR Trends for Regional Suicide Mortality Ratios 

**Figure 2:**
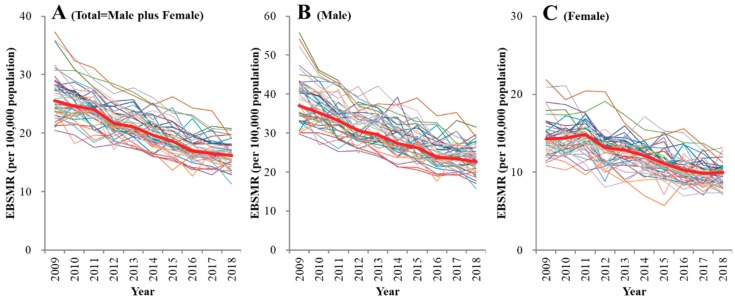
Trends of empirical Bayes standardized mobile ratio (EBSMR) of suicide mortality per 100,000 population between 2009 and 2018 for (**A**) total (male plus female), (**B**) males and (**C**) females in 47 Japanese prefectures, and national average (red). Ordinates: EBSMR of suicide mortality for each prefecture (per 100,000 population); abscissa: time (year).

There was a statistical reduction in EBSMR for the total (male plus female) and male suicide mortality for all prefectures from 2009 to 2018 (*p* < 0.05) (Figure 2A,B). In addition, there was also a reduction in EBSMR for female suicide mortality during the same period (*p* < 0.05), with the exception of two prefectures, including Mie, which showed no significant change (Figure 2C).

**Figure 3:**
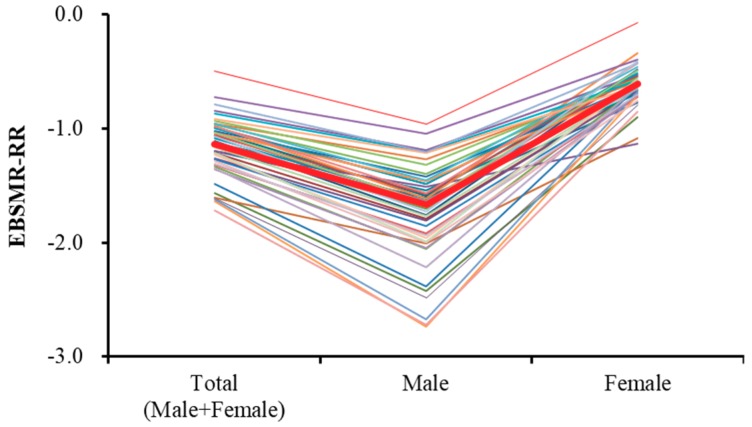
Time-dependent trends of EBSMR (EBSMR-RR) of total (male plus female), male and female suicide mortality in 47 Japanese prefectures, including national average (red) between 2009 and 2018 calculated while using the least squares method.

Time-dependent reduction trends of total EBSMR of suicide mortality (EBSMR-RR) for all 47 prefectures are mean ± SD = −1.15 ± 0.26 and median = −1.13 (Figure 3). The EBSMR-RR of males for all 47 prefectures are mean ± SD = −1.74 ± 0.43 and median = −1.63 (Figure 3). The EBSMR-RR of females for all 47 prefectures are mean ± SD = −0.61 ± 0.18 and median = −0.62 (Figure 3). Therefore, the EBSMR of total, male, and female suicide mortality decreased 1.15, 1.74, and 0.61 per year, respectively. 

### 3.2. Relationship between Suicide Mortality Trends (EBSMR-RR) and REA in 47 Prefectures 

**Figure 4:**
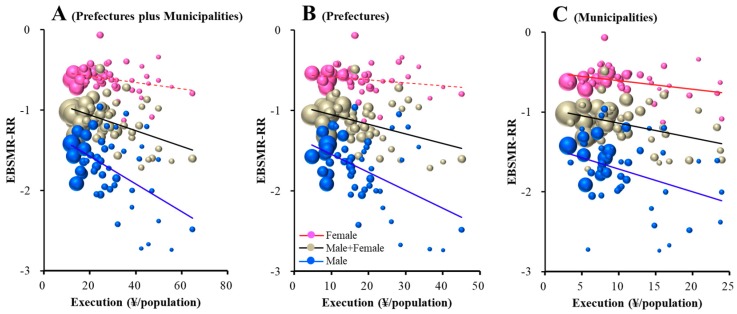
Relationship between EBSMR-RR and regional execution amounts (REAs) in 47 prefectures: (**A**) prefectures plus municipalities, (**B**) prefectures and (**C**) municipalities. Ordinates indicate EBSMR-RR and abscissas indicate REAs (¥ per population). Sphere size indicates the population size of each prefecture. Brown, blue and red indicate EBSMR-RR of total (male plus female), male and female, respectively. Linear regression analysis indicates total EBSMR-RR = −0.010 × REA (prefectures plus municipalities) − 0.866, = −0.012 × REA (prefectures) − 0.932, and = −0.018 × REA (municipalities) − 0.987. Male EBSMR-RR = −0.017 × REA (prefectures plus municipalities) − 1.231, = −0.022 × REA (prefectures) − 1.321, and = −0.028 × REA (municipalities) − 1.432. Female EBSMR-RR = −0.011 × REA (municipalities) − 0.498.

The total EBSMR-RR was significantly/inversely related to REAs of prefectures plus municipalities, prefectures, and municipalities (Figure 4). Leaner regression analysis results suggest that ¥10.2, 8.1, and 5.5 million per 100,000 population for prefectural plus municipal, prefectural, and municipal REAs, respectively, for regional suicide prevention decrease total EBSMR by one per year. The EBSMR-RR of males was also significantly/inversely related to REAs of prefectures plus municipalities, prefectures, and municipalities (Figure 4). Leaner regression analysis results suggest that ¥5.8, 4.5, and 3.5 million per 100,000 population for prefectural plus municipal, prefectural, and municipal REAs, respectively, for regional programmes decrease EBSMR for males by one per year. However, the EBSMR-RR of females was significantly/inversely related to REAs of municipalities, but not prefectures plus municipalities or prefectures (Figure 4). Leaner regression analysis results suggest that ¥9.3 million per 100,000 population of municipal REAs for regional programmes decrease EBSMR for females by one per year. Therefore, REAs of prefectures plus municipalities, prefectures and municipalities showed a size-dependent reduction in the total and male suicide mortality, without affecting female mortality. However, municipal REAs showed a size-dependent reduction in female suicide mortality.

### 3.3. Relationship between EBSMR-RR and 10 REA Subgroups 

Stepwise multiple regression analyses were conducted to predict the total (male plus female), male, and female EBSMR-RR according to the 10 sub-divisions of REAs, including prefectural and municipal “personal consultation support”, “telephone consultation support”, “development of listeners and leaders”, “enlightenment”, and “intervention models” programmes. Table 1 summarizes the results of the analysis.

**Figure 5:**
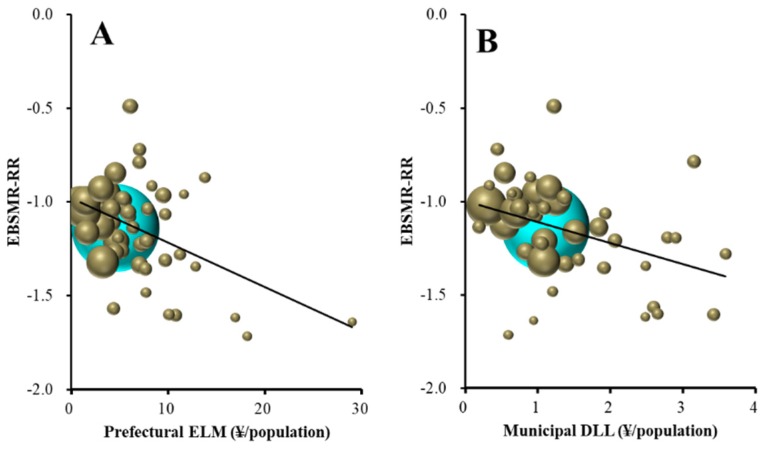
Relationship between total (male plus female) EBSMR-RR and sub-divisions of REAs: (**A**) prefectural enlightenment programmes and (**B**) municipal development programmes of leaders and listeners. Ordinates indicate total EBSMR-RR and abscissas indicate sub-divisions of REAs (¥ per population). Sphere size indicates the population size of each prefecture. Light blue spheres indicate the population size of the nation. Total EBSMR-RR = −0.021 × (prefectural ELM) − 0.092 × (municipal DLL) − 0.873. There was no multicollinearity.

Stepwise multiple regression analysis analysed the relationship between total EBSMR-RR and the 10 sub-divisions of RAEs. Total EBSMR-RR was significantly/inversely related to prefectural enlightenment programmes and municipal development of listeners and leaders (Table 1 and Figure 5). Multiple regression analysis results suggest that ¥4.8 million per 100,000 population for prefectural enlightenment programmes and ¥1.1 million per 100,000 population for municipal development programmes of leaders and listeners for regional suicide prevention decrease total EBSMR by one per year.

**Figure 6:**
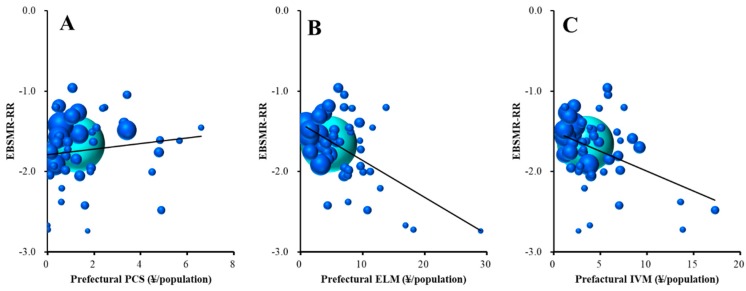
Relationship between male EBSMR-RR and sub-divisions of REAs: (**A**) prefectural personal consultation support programmes, (**B**) enlightenment programmes, and (**C**) intervention model programmes. Ordinates indicate male EBSMR-RR and abscissas indicate sub-divisions of REAs (¥ per population). Sphere size indicates the population size of each prefecture. Light blue spheres indicate the population size of the nation. Male EBSMR-RR = +0.065 × (prefectural PCS) − 0.043 × (prefectural ELM) − 0.039 × (prefectural IVM) − 1.347. There was no multicollinearity.

The male EBSMR-RR was significantly/inversely related to prefectural enlightenment programmes and intervention models (Table 1, Figure 6B,C); however, surprisingly, it was significantly/directly related to prefectural personal consultation support (Table 1 and Figure 6A). The multiple regression analysis results suggest that ¥2.3 million per 100,000 population for prefectural enlightenment programmes and ¥2.6 million per 100,000 for intervention model programmes for regional suicide prevention decrease EBSMR for males by one per year. On the contrary, ¥1.5 million per 100,000 population for prefectural personal consultation support programmes for regional suicide prevention increases one/year of male EBSMR.

**Figure 7:**
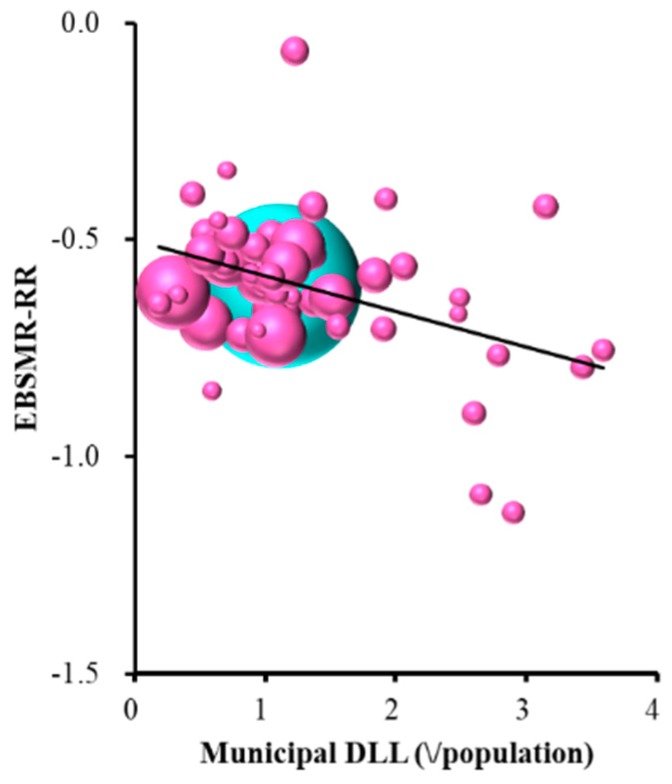
Relationship between female EBSMR-RR and ELM sub-division of REAs. Ordinate indicates female EBSMR-RR and abscissa indicates sub-division of REAs (¥ per population). Sphere size indicates the population size of each prefecture. Light blue spheres indicate the population size of the nation. Female EBSMR-RR = −0.083 × (municipal DLL) − 0.500. There was no multicollinearity.

Female EBSMR-RR was significantly and inversely related to municipal development of listeners and leaders (Table 1 and Figure 7). The multiple regression analysis results suggest that ¥1.2 million per 100,000 population for municipal development programmes of leaders and listeners for regional suicide prevention decrease EBSMR for females by one per year.

## 4. Discussion

This study demonstrates the important contribution of governmental financial support through the ‘Emergency Fund to Enhance Community-Based Suicide Countermeasures’ to the reduction in the national suicide mortality rates. In addition, this study identifies the different contributions of EBSMR-RR of males and females to REAs. REA size-dependently reduces the suicide mortality rates. In general, prefectural REAs were found to improve the EBSMR-RR of males, but not females, whereas municipal REAs improved both (Figure 4). These results may be due to the interaction between socioeconomically gender-specific factors. The gender-specific analysis suggests that the prefectural REAs for enlightenment and intervention model programmes improve the EBSMR-RR of males without affecting that of females, while EBSMR-RR of females is specifically improved by municipal REAs for the development of listeners and leaders. 

The different sensitivities of EBSMR-RR of males and females to financial support for regional suicide prevention programmes is, at least partially, explained by the gender-specific socioeconomic background of suicide pathology. Corporate restructuring might cause the higher suicide mortality rate of middle-aged Japanese male [20]. Indeed, employment insecurity and the lack of social protection in economically active environments are associated with high suicide mortality rates amongst middle-aged Japanese male [21]. In other words, not only unemployment, but also the fear of losing one’s job may be a contributing factor to male suicide [20]. Indeed, over the past two decades, an increase in suicide mortality amongst middle-aged male due to modifications in the corporate environment have been observed in other Asian countries, such as South Korea, Hong Kong, and Taiwan [8]. Taken together with other research findings, the results of this research point to the importance of enriching prefectural social community support (including enlightenment programmes and intervention models) to prevent an increase in male suicide mortality. An unexpected finding of the research was that prefectural personal consultation support negatively affects the EBSMP-RR of males. This paper does not explore the detailed reasons for this, but it might be be that personal consultation is a limited prevention method in the case of male suicide, as it cannot rectify an individual’s economic or employment situation.

A feature of Japanese social pathology likely causes the effectiveness of municipal development of listeners and leaders against selectively female suicide prevention. Although Japanese women have experienced some social advancement, they continue to have primary responsibility for childcare in a social system that offers poor childcare support. Most women are forced to play a subordinate role, due to limited childcare support resources, leading to temporary employment interruptions and a decreasing social circle due to the demands of childcare. In addition, Japan’s declining birth rate and aging population have come to resemble the social conditions of China’s one-child policy [22,23,24]. Therefore, if the pressure to bear and rear children is a factor in female suicide, then improvements in childcare support resources and finding satisfaction in a relatively small childcare community might contribute to a reduction in female suicide mortality rates. 

Cost-effectiveness and an evaluation of the success of REAs are both required for the development of evidence-based regional suicide prevention programmes; however, this study analysed quantitative optimisation of REAs, but it did not undertake a qualitative evaluation of REAs. Japan’s community-based suicide prevention programmes show encouraging results by connecting service providers with individuals in need [25,26]. It is also well established outside of Japan that effective community-based programmes contribute to reduced regional suicide mortality rates [20]. These previous reports suggest that socioeconomic transition and withdrawal from support systems both increase the risk of suicide [20]. Therefore, an evaluation of effectiveness regarding governmental support for the regional intervention model is needed, as to whether it does develop functional community-based suicide prevention programmes.

Finally, the suicide mortality rate in Japan has been reduced by the government’s comprehensive prevention programme, including accruing knowledge from the latest suicide prevention research, introducing a system for the early detection of high-risk individuals, targeting medical service delivery to such individuals, and providing support for suicide attempt survivors. The findings of this research indicate the importance of government financial support for regional suicide prevention programmes, with special emphasis on the importance of the quantitative distribution of funding for the reduction of regional suicide mortality (EBSMR-RR). 

There are several limitations to this study. No covariates other than age and gender were used for confounder adjustment. The data were not disaggregated by municipality, which might provide more detailed information regarding the role of REAs in regional suicide prevention programmes and aging in the change in level. We also did not consider other factors, such as trends in GDP, workforce structure, or unemployment [27,28], which might further explain the role of REAs in the overall decrease in suicide mortality. Having a detailed understanding of suicide mortality based on the age structure in an aging country, such as Japan, and identifying specific innovations in suicide prevention programmes are essential for effective suicide prevention in the future. Nonetheless, the general downward trend in suicide mortality in this study suggests that progress is being made on a comprehensive prevention programme in Japan. However, further interventions in the 40- to 79-year-old age group are necessary to further reduce the major causes of suicide mortality in Japan (Figure 1A). Regional public health planners and policymakers in the prefectural and municipal offices should consider specific actions to reduce regional suicide mortality as a method of suicide prevention in Japan by identifying the social drivers, such as personal consultation support programmes, telephone consultation support programmes, development programmes of leader and listener, enlightenment programmes, and intervention model programmes. In the future, the budget structure should be improved to be more cost-effective in the prevention of suicide and, following several observations of suicide mortality rates in Mie Prefecture, the importance of a budget structure for suicide prevention should be reported.

## 5. Conclusions

The present study indicates the possibility that governmental financial support in Japan contributes to a reduction of suicide mortality rates. Total EBSMR-RR (male plus female) was significantly/inversely related to prefectural ‘enlightenment’ and the municipal ‘development of listeners and leaders’. The EBSMR-RR of males was significantly/inversely related to prefectural ‘enlightenment programmes’ and ‘intervention models programmes’ but significantly/directly associated with prefectural ‘personal consultation support programmes’. EBSMR-RR of females, on the other hand, was only significantly/inversely related to the municipal ‘development programmes of listeners and leaders’. These present findings provide important information for planning a scientifically evidence-based and more cost-effective suicide prevention programme.

## Figures and Tables

**Table 1:** Stepwise multiple regression analysis of EBSMR-RR and 10 sub-divisions of RAEs: personal consultation support (PCS), telephone consultation support (TCS), development of leader and listener (DLL), enlightenment (ELM), and intervention model (IVM) programmes. β means standard partial regression coefficient. Multicollinearity was suspected if the variance inflation factor (VIF) value was greater than 10.

Variables	Total (Male plus Female)			Male			Female		
	*β*	*p*	VIF	*β*	*p*	VIF	*β*	*p*	VIF
**Prefecture**									
PCS				+0.252	<0.05	1.04			
TCS									
DLL									
ELM	−0.410	<0.01	1.03	−0.500	<0.01	1.08			
IVM				−0.312	<0.05	1.08			
**Municipality**									
PCS									
TCS									
DLL	−0.312	<0.05	1.03				−0.409	<0.01	1.00
ELM									
IVM									
	F = 9.806 Adjusted R^2^ = 0.277	<0.01		F = 10.697 Adjusted R^2^ = 0.389	<0.01		F = 9.066 Adjusted R^2^ = 0.149	<0.01	
